# Editorial: Exercise and neuroplasticity in Parkinson disease

**DOI:** 10.3389/fneur.2025.1587715

**Published:** 2025-03-24

**Authors:** Mark A. Hirsch, Ulrik Dalgas, Erwin E. H. van Wegen

**Affiliations:** ^1^Carolinas Rehabilitation, Department of Physical Medicine and Rehabilitation, Charlotte, NC, United States; ^2^Wake Forest University School of Medicine, Winston-Salem, NC, United States; ^3^Exercise Biology, Department of Public Health, Aarhus University, Aarhus, Denmark; ^4^Amsterdam University Medical Center, Department of Rehabilitation Medicine, Amsterdam Movement Sciences, Amsterdam, Netherlands

**Keywords:** Parkinson's disease, exercise, neuroplasticity, biomarker, rehabilitation

Historically, exercise was believed to be a waste of time, or even harmful, by increasing the amount of underlying muscle tone. Little research was conducted. Consequently, limited progress was made in our understanding of the interplay between exercise and the degenerative brain process in rehabilitation of individuals living with Parkinson's disease (PD). In the last decade, epidemiologic evidence has established an association between the amount of physical activity and risk of PD (primary prevention) (1); supports a disease-modifying secondary prevention role (2); provides plausible rationale of exercise for symptomatic relief (i.e., tertiary prevention), highlighting the pivotal role of exercise (2).

Although a neuroprotective or neurorestorative disease-altering role of exercise for individuals at all stages of PD remains controversial and mysterious (3, 4), an inflection point has occurred that will lead us into a new generation.

## We are entering a new era

Growth in the number of scientific studies on effect of exercise on PD clinical progression (5–7), as assessed in the off-medication state by the widely used Movement Disorders Society-Unified Parkinson's Disease Rating Scale (MDS-UPDRS motor section), has been noted over the past decade. Frazzitta et al. (8) were among the first to use a randomized controlled trial design to hypothesize an interplay between intensive physical exercise and endogenous production of brain-derived neurotrophic factor, with positive effect on MDS-UPDRS, in the rehabilitation of human PD. Meta-analytic evidence supports this hypothesis (9, 10).

Using a case study design of a single runner with PD assessed by DaTSCAN (Ioflupane i 123) 3 days before and 3 days after running a 100 km ultramarathon race, Daviet et al. (11) reported longitudinal improvement in bilateral posterior putamen-caudate nucleus dopamine transporter (DAT) binding (DAT is a protein critical in maintaining intracellular dopamine storage in the substantia nigra). Interestingly, following the race, the authors point out that the patient was able to eliminate his levodopa pills and benserazide, a peripheral decarboxylase inhibitor that increases the amount of levodopa crossing the blood-brain barrier and its subsequent conversion to dopamine. Similarly, in 2013, Fisher et al. reported elevated striatal dopamine D2 receptor binding potential after treadmill exercise in 2 patients with early PD (12). Using data from the Parkinson Progressive Markers Initiative, Shih et al. reported a positive significant mediating effect of physical activity in the relationship between DAT in the caudate and putamen separately and PD global cognition (13). Sacheli et al. reported exercise-induced increase in caudate dopamine release and ventral striatal activation as measured by positron emission tomography in participants with mild to moderate idiopathic PD after 3 months of aerobic cycling using a stationary ergometer (14). Using resting state and functional MRI Johansson et al. found remotely supervised exercise involving cycling on a stationary bike with “exergaming” features 3 days per week over the 6-month course of the Park-in-Shape aerobic exercise trial increased cognitive control, functional connectivity of the anterior putamen with the sensorymotor cortex, right fronto-parietal network, and reduced global brain atrophy, possibly stabilizing disease progression (15). Finally, de Laat et al. (16) evaluated the effects of a 6-month high-intensity interval training program mirroring the ParkFit exercise program (17) in 13 high functioning participants with mild bilateral PD with intact cognition and no significant mood disturbances. Using PET in an uncontrolled proof-of-concept design with MRI assessment for neuromelanin (a hallmark of PD pathology), the authors report exercise-induced “reversal [of an] expected decrease in DAT availability in substantia nigra and putamen” (16). In addition, a significant increase in the neuromelanin signal in the substantia nigra was observed, which was interpreted by the authors as likely reflecting “improved metabolic/synthetic functionality in the remaining dopaminergic neurons post-exercise” (16). Taken together, the evidence on exercise-induced neuroplastic effects and its underlying mechanisms in people with PD is in its early infancy but does point in a positive direction.

The current Research Topic adds to this positive direction with research and scholarship on exercise and neuroplasticity among 259 participants with PD by five research teams in 3 countries:

San Martin Valenzuela et al. provide a randomized controlled trial protocol to evaluate markers of motor imagery to improve PD gait among 88 participants with idiopathic PD.

Rotondo et al. employ a pilot, prospective, observational cohort study design to evaluate dosing effects of exercise on peripheral biomarkers, neurotrophins, Insulin-like growth factor-1 or Irisin, and possible downstream neuroplasticity brain connectivity mechanisms among 30 participants with mild to moderate PD.

Lombardi et al. provide a randomized controlled trial protocol to evaluate the effect of intensive treadmill training on prognostic biomarkers associated with blood-derived extracellular vesicles among 48 participants with mild to moderate PD with gait disturbance.

Luo et al. provide a trial protocol to assess the effect of Yijnjing exercise on prefrontal cerebral and sensorimotor cortex blood oxygen signal level among 96 eligible participants with PD.

Zikerya et al. review evidence supporting dopamine depletion as the basis for dysfunctional goal-directed and habitual basal ganglia control circuitry.

“Thank you” to these teams for reminding us that optimal exercise prescription can only be obtained if we look more deeply and think beyond motor function.

## We need to make this a “we” journey and not a “me” journey

We emphasize that exercise-neuroplasticity is a young science still in its early infancy, very open to exploration. Much still remains to be discovered and we need to work together and remain humble. How will we get to new therapies? We don't understand the critical steps at play in PD. Can the appropriate exercise regimen slow or perhaps even halt disease progression? Big questions cannot be answered by individuals. Increasingly, a team science approach is adopted. We hope the current Research Topic promotes a more radical team science approach, greater collaboration among patient-scientist-clinician in which people with PD are empowered as “partners” or “colleagues” rather than mere “objects” or “subjects” of research. We need hard work to gain insight into the potential of physical exercise as a disease modifying treatment and not handwaving about the degenerative process. So how about we get busy!
